# Exposure assessment of dairy cows to parabens using hair samples analysis

**DOI:** 10.1038/s41598-024-65347-z

**Published:** 2024-06-21

**Authors:** Slawomir Gonkowski, Manolis Tzatzarakis, Nariste Kadyralieva, Elena Vakonaki, Thomas Lamprakis

**Affiliations:** 1 Department of Clinical Physiology, Faculty of Veterinary Medicine, University of Warmia and Mazury in Olsztyn, Oczapowskiego 13, 10-957 Olsztyn, Poland; 2 Laboratory of Toxicology, School of Medicine, University of Crete, 71003 Heraklion, Crete Greece; 3 Department of Histology and Embryology, Veterinary Faculty, Kyrgyz-Turkish Manas University, Bishkek, Kyrgyzstan

**Keywords:** Biomonitoring, Methyl paraben (MeP), Farm animals, Central Asia, LC–MS method, Ecology, Environmental sciences, Risk factors

## Abstract

Parabens (PBs) are used as preservatives in various products. They pollute the environment and penetrate living organisms, showing endocrine disrupting activity. Till now studies on long-term exposure of farm animals to PBs have not been performed. Among matrices using in PBs biomonitoring hair samples are becoming more and more important. During this study concentration levels of methyl paraben (MeP), ethyl paraben (EtP), propyl paraben (PrP) butyl paraben (BuP) and benzyl paraben (BeP) were evaluated using liquid chromatography–mass spectrometry (LC–MS) in hair samples collected from dairy cows bred in the Kyrgyz Republic. MeP was noted in 93.8% of samples (with mean concentration levels 62.2 ± 61.8 pg/mg), PrP in 16.7% of samples (12.4 ± 6.5 pg/mg) and EtP in 8.3% of samples (21.4 ± 11.9 pg/mg). BuP was found only in one sample (2.1%) and BeP was not detected in any sample included in the study. Some differences in MeP concentration levels in the hair samples depending on district, where cows were bred were noted. This study has shown that among PBs, dairy cows are exposed mainly to MeP, and hair samples may be a suitable matrix for research on PBs levels in farm animals.

## Introduction

Nowadays, farm animals are exposed to many harmful substances, that pollute the natural environment^1,2^. These substances cause health disorders in animals, penetrate products of animal origin and pose a potential threat to humans^3^. A group of such substances, that commonly pollute the environment and are easily absorbed into living organisms are parabens (PBs)^4^.

PBs are organic substances, that are chemically the alkyl esters of parahydroxybenzoic acid^5^. Individual PBs differ from each other by the type of substituent, which may have a form of an alkyl chain or an aromatic ring^4^. In the nature PBs are produced by some species of microbes and plants^6^. However, the majority of PBs found in the natural environment are of anthropogenic origin^4,7^. PBs have been synthesized and used on a large scale in various industries since the 1930s^4,7^. It is connected with their anti-fungal, anti-yest, anti-molt and (slightly weaker) anti-bacterial activities^7^. Due to these properties, PBs are used in the industry as preservatives, i.e. ingredients that inhibit the growth of microorganisms and extend the shelf life of products^4,7^. Nowadays, PBs are commonly added to various types of cosmetics, personal care products, preserved food and food containers, pharmaceuticals and many other products^5,7^. The most popular PBs used in the industry are methyl paraben (MeP), ethyl paraben (EtP), propyl paraben (PrP) butyl paraben (BuP) and benzyl paraben (BeP)^4,7^.

PBs may penetrate the natural environment, and due to their widespread use in the industry, they are often found in surface and tap water, soil, air and dust on all continents, even in Antarctica^7–9^. Previous studies have also proven that PBs may relatively easily penetrate the human and animal organisms through the gastrointestinal tract, respiratory system, skin and (in prenatal period) placenta^4,7,10^. Until recently, PBs were believed to be harmless to humans and animals^11^. However, more recent research has proven that these substances have harmful effects. It has been shown that PBs show endocrine disrupting properties and may, among others, adversely affect the endocrine, reproductive, immune and nervous systems^7,12,13^. Moreover, some studies have indicated genotoxic and cytotoxic activity of PBs^4,14^, as well as the connections between exposure to these substances and risk of diabetes, hypertension, allergic reactions and degenerative processes^7,15,16^. However, it should be pointed out that toxic properties of PBs are not fully known and are still controversial^11^. In the light of these findings, biomonitoring of PBs seems to be an important issue in the environmental toxicology.

The vast majority of previous works describing PBs in living organisms concern humans and wild water animals^7,17–19^. The presence of PBs has been confirmed in people from different parts of the world not only in matrices typical for biomonitoring studies, such as blood serum or urine^17,20^, but also in many other matrices, including breast milk^21^, semen^22^, amniotic fluid^23^ and hair^24^. In turn in animals, fragments of liver, kidneys or muscles have been most often studied for the presence of PBs^8,25,26^. Among these matrices the hair samples seem particularly interesting. The concentration levels of substances accumulated in the hair (unlike blood serum or urine) do not fluctuate in the short term^27^. Therefore, the hair samples seem to be the optimal matrix for long-term environmental exposure studies^27,28^. Moreover, sample collection, storage and transport are easy, and (as previous studies have shown) results obtained during hair samples analysis have similar sensitivity and repeatability to those obtained during the analysis of typical matrices, such as blood serum or urine^27–29^.

It should be underlined that knowledge of domestic animals’ exposure to PBs is extremely scanty. In dogs and cats, living in close proximity to humans PBs have been studied in the urine^30^ and hair samples^31^. In the case of farm animals, not numerous previous studies focus on PBs concentration levels in products of animal origin, such as milk or meat^32–34^, as well as commercially available cow urine distillate, which is regarded as a drug for many diseases in India^35^. According to the best knowledge of the authors, till now there are no studies focusing exclusively on the long-term exposure of farm animals to PBs. On the other hand the assessment of such exposure would be very helpful in the risk evaluation connected with PBs in an environment, where animals live for both animal health and the safety consumers of animal products.

Taking this into account, the aim of the present study was to investigate the degree of dairy cows’ exposure to selected, the most common BPs (i.e. MeP, EtP, PrP, BuP and BeP) through analysis of the hair samples collected in various regions localized around Bishkek—the capital and biggest city of the Kyrgyz Republic. This is not only the first study on long-term exposure to PBs in dairy cows, but also the first investigation concerning PBs in the Kyrgyz Republic. Therefore results obtained in the present work clearly expand knowledge about the presence of PBs in the environment and in the bodies of farm animals.

## Results

Among the substances covered by the research, MeP was the most frequently observed (Table 1). It was found in above 90% of hair samples included into the study. Only in one sample MeP was below LOQ, and in 3 samples MeP was not detected (Supplementary Materials—Table S1). In other samples MeP levels ranged from 12.0 to even above 360 pg/mg with a mean 62.2 ± 61.8 pg/mg. Clear differences in MeP concentration levels were visible between particular animals, even between cows raised in the same area (Supplementary Materials—Table S1).

**Table 1:** Concentration values (pg/mg) and frequency of detection (%) of parabens (n = 48)—cumulative data.

Compound	Min*	Max*	Mean ± SD*	Median*	Frequency of detection
MeP	12.0	365.5	62.2 ± 61.8	46.7	93.8
EtP	13.0	38.8	21.4 ± 11.9	16.8	8.3
PrP	9.1	27.1	12.4 ± 6.5	10.1	16.7
BeP	–	–	–	–	0
BuP	–	–	–	–	2.1

*MeP* methyl paraben, *EtP* ethyl paraben, *PrP* propyl paraben, *BeP* benzyl paraben, *BuP* butyl paraben.

*Only values above LOQ were included.

Apart from MeP, EtP, PrP and BuP were also found in the hair samples during this investigation, but their frequency of occurrence was much lower. Namely PrP was noted in 16.7% of all samples, and EtP in 8.3%. PrP and EtP concentration levels were also significantly lower than MeP. Maximum concentration level noted during this study did not exceed 40 pg/mg in the case of EtP and 30 pg/mg in the case of PrP. Median values of EtP and PrP amounted to 21.4 ± 11.9 pg/mg and 12.4 ± 6.5 pg/mg, respectively. In turn, BuP was found only in one sample (2.1% of all samples analyzed) at a concentration of 7.6 pg/mg (Supplementary Materials—Table S1). BeP was not detected in any sample included into study. Results obtained in the present study are summarized in Table 1.

**Figure 1:**
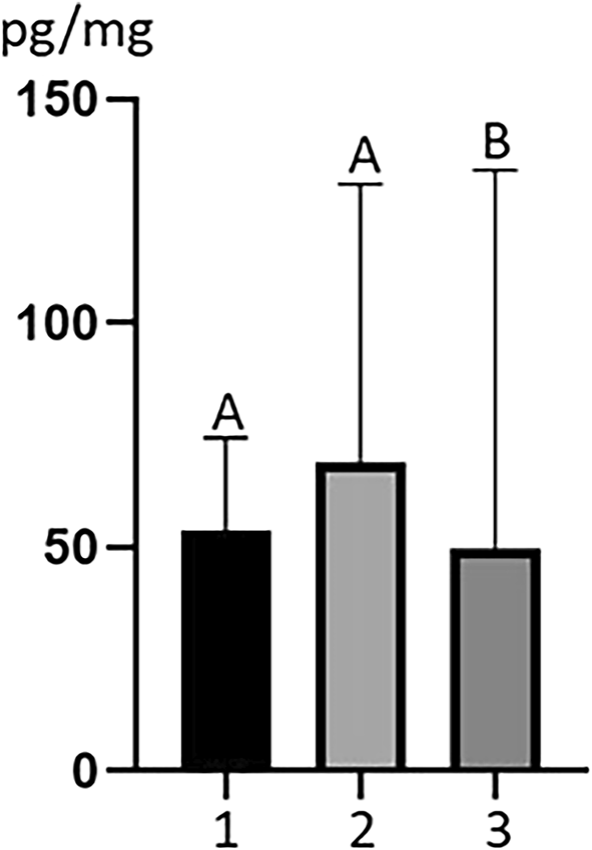
Mean concentration levels (± SD) of methyl paraben (MEP) in dairy cow hair samples in Sokuluk (1), Alamedin (2) and Ysyk Ata (3) districts. Statistically significant values (*p* < 0.05) are indicated with various letter, and values statistically insignificant are indicated with the same letter.

The highest mean concentration level of MeP was found in the Alamedin district, where this value amounted to 68.95 ± 62.13 pg/mg (Fig. 1). A mean concentration level of this substance in the Sokuluk district was slightly lower and amounted to 53.81 ± 20.64 pg/mg. However, differences in MeP concentration levels between Alamedin and Sokuluk districts were not statistically significant. The lowest mean concentration level of MeP was noted in the Ysyk Ata district. In this district mean concentration level of MeP achieved 49.71 ± 84.44 pg/mg and was statistically significantly lower than those observed both in Alamedin and Sokuluk districts (Fig. 1).

**Figure 2:**
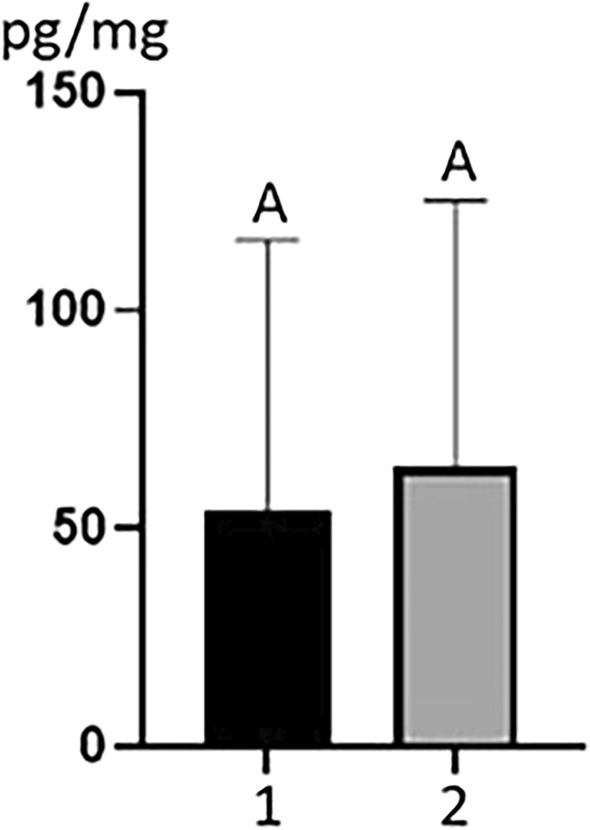
Mean concentration levels (± SD) of methyl paraben (MEP) in the hair samples of dairy cows at the age 3–4 years (1) and 5–8 years (2). Statistical insignificant values (*p* < 0.05) are indicated with the same letter.

During the present study mean concentration levels of MeP in animals of different ages were also compared (Fig. 2). In younger animals (at the age of 3–4 years) mean concentration level of MeP amounted to 53.95 ± 62.26 pg/mg, while in older animals (at the age of 5–8 years old) this value was slightly higher and amounted to 64.07 ± 61.29 pg/mg (Fig. 2). However, differences between these values were not statistically significant. Other PBs were found in too few samples to compare their concentrations in animals from different regions or in animals of different ages.

## Discussion

This is the first study on the exposure of farm animals to PBs through analysis of the hair samples. Till now this matrix has been used only to determine of PBs concentration levels in humans and dogs (Table 2).

**Table 2:** The levels of parabens in hair samples (pg/mg) noted in previous studies.

Country	n	MeP	EtP	PrP	BeP	BuP	Ref.
Humans							
Belgium	114	Range: < LOQ-53356.0 Median: 28.9	Range: < LOQ-26967.7 Median: 5.2	Range: < LOQ-1517.1 Median: 16.4			^36^
France	5	Range: 0.37–318.54 Mean: 69.74 ± 139.54	Range: < LOQ-1.41	Range: < LOQ-291.74		Range: < LOQ-0.874	^37^
Germany	4		Range: 810–1980	Range: 400–1520			^38^
Greece	95	Range: 17.6–27,437.0 Median 1437.1 Mean: 4501.2 ± 6756.1	Range: 11.0–4224.5 Median 167.3 Mean: 510.1 ± 857.6		Range: 2.1–66.6 Median: 15.9 Mean: 22.9 ± 24.1	Range: 1.8–2513.7 Median: 78.6 Mean: 237.1 ± 422.3	^39^
Korea	10	Range: 48.3–224.2 Mean: 123.6 ± 61.6	Range: 11.5–158.3 Mean: 64.5 ± 43.5	Range: 70.2–214.5 Mean: 136.9 ± 48.5	Range: 15.3–100.2 Mean: 55.6 ± 24.3	Range: 25.4–111.1 Mean: 74.2 ± 27.5	^40^
Poland	30	Range: 87.2–42,430.1 Median: 604 Mean: 4302.0 ± 10,281.8	Range: 19.1–8413.1 Median: 120.7 Mean: 704.0 ± 1775.4	Range: 18.9–11,150.3 Median: 169.5 Mean: 825.7 ± 2166.0	Range: 36.0–700.7 Median: 87.2 Mean: 135.2 ± 144.5	Range: 3.8–2636.0 Median: 27.8 Mean: 154.5 ± 501.1	^24^
Spain	6	Range: 78–624	Range: 7.0–42	Range: 27–238			^41^
	6	Range: 10.2–33	Range: 9.0	Range: 11.6–107		Range: 3.5–9.4	^42^
	42	Range: 68.3–14,187 Median: 822.1 Mean: 2820.7	Range: 2.9–6565 Median: 47.2 Mean: 634.8	Range: 12.5–9009 Median: 256.3 Mean: 1006.1			^42^
DOGS							
Poland	30	Range: < LOQ-1023 Median: 118 Mean: 176	Range: < LOQ-382 Median: 17.9 Mean: 48.4	Range: 8.12–527 Median: 39.4 Mean: 79.8			^31^

*LOQ* limit of quantification, *n* number of samples.

Such limitation is somewhat surprising, because the hair as a matrix to biomonitoring studies has undeniable advantages. First of all the hair samples seem to be optimal for studies on long-term exposure to the environmental pollutants. It has been shown that “detection window” i.e. the time from the moment of exposure to the possibility of detecting a given substance in the body in the case of hair analysis can be months or even years, depending on the length of hair, while during blood serum or urine analysis “detection window” is measured usually in hours or days^28,43^. This fact seems to be especially important in biomonitoring studies on substances rapidly eliminated from organism (which include among others PBs), for which blood serum or urine samples analysis reflects recent exposure and is characterized by high fluctuations in rapid succession^44–46^.

Moreover, the undoubted advantages of hair as a matrix are easy, painless and stress-free collection of samples (which is particularly important in animals, especially skittish or aggressive ones) and easy sample storage. Of course, analysis of the hair samples has some limitations. Substances can penetrate the hair both from the inside, reaching the hair roots through capillaries, and directly from the external environment, and it is impossible to separate these two sources of origin during sample analysis^27,45^. Moreover, the hair samples are not suitable for studies on factors connected with short-term exposure to substances in the environment. Nevertheless, the advantages of the hair samples support the wider use of this matrix for biomonitoring research on PBs, especially since the results of the present study have proven the possibility of such using in farm animals.

Comparing previous results with those obtained in this study, it can be concluded that generally PBs in humans and dogs have been detected at a higher frequency and in higher concentrations than in cows (Table 2). Such situation may result from the fact that PBs are typical anthropogenic pollutants of the environment. Therefore, the highest degree of exposure to PBs concerns humans and companion animals, living in close proximity to people in the same environment^17,29,31^. In companion animals relatively high PBs levels have been noted not only in the hair samples (dogs)^31^, but also in the urine (dogs and cats)^30^. It should be pointed out that the exposure to PBs depends not only on industrialization or urbanization of a given area, but also on various other factors, which are irrelevant in the case of farm animals. In the light of previous studies, these factors may include for example the workplace, lifestyle, preserved food and drinks or the frequency of using cosmetics and personal care products^47^. From this reason the PBs concentration levels noted in the present study are lower than those noted in humans and dogs (Table 2). On the other hand, farm animals, through the contact with humans, are also exposed to anthropogenic pollutants. which is confirmed by the fact that PBs concentration levels (especially MeP) noted in the present study are generally higher than values observed in wild animals living especially in less urbanized areas^8,18,48^. However, PBs are also present in rural areas^49^. The important source of human and animal exposure to PBs in rural regions is connected with the fact that various environmental pollutants, including PBs, are discharged into sewage systems and with the sewage they penetrate into the environment through surface water and groundwater. Previous studies have indicated the presence of PBs both in the sewage^50,51^ and in the surface waters^52^. Various pollutants may also penetrate into the rural environment with water used to the crop irrigation^53^. This is all the more likely that nowadays the waste valorization in the agriculture is increasingly used. The irrigation of crops with reclaimed wastewater or soil fertilization with sewage sludge may result in the increase in pollution of the soil with various anthropogenic substances (including PBs) and cause the risk of absorption of these substances by arable crops^54^. Probably this fact may also influence on the degree of farm animal exposure to PBs, although till now such investigations have not been performed.

A wide range of factors influencing environmental pollution with PBs means that the levels of these compounds vary drastically in different parts of the world (Table 2). In the light of previous investigations one of the main factors influencing the level of PBs in the environment is urbanization and industrialization^17,55^. Unfortunately the evaluation of the influence of these factors on the present result is impossible, because till now there were no investigations concerning PBs in the environment in the Kyrgyz Republic. However, the impact of urbanization and industrialization may be manifested in the fact that MeP concentration levels in the cow hair were the lowest in the Ysyk Ata district, which is the furthest from Bishkek among the regions covered by this study. It should be noted that Bishkek is the largest city in Kyrgyzstan with over a million inhabitants and a large industrial center with high levels of pollution with anthropogenic substances^56,57^. Moreover, the highest concentration levels of MeP was noted in the Alamedin district, in which number of villages was higher than in other district included into the study. However, it cannot be excluded that mentioned above factors contributing to PBs presence in the rural environment significantly influenced the obtained results.

Results obtained in the present study have shown that MeP plays the most important role in the exposure of cows to PBs. It is in agreement with previous observations, that have shown that MeP, among all parabens, occurs most often and in the largest amounts both in the natural environment and living organisms^17,24,31^. This fact applies to various regions of the world, what may indicate that environmental pollution with MeP is a global problem resulting from the widespread use of short-chain PBs in various branches of the industry.

It should be pointed out, that according to the best knowledge of the authors only two previous studies concern PBs concentration levels in cows. In the first of them PBs have been found in cow urine distillate, which is regarded as a drug for many diseases in India^35^. In cow urine distillate MeP, EtP, BuP and BeP have been detected in relatively high number of samples (from about 30% for BeP to about 95% for MeP) and high mean concentration levels (for example 961.81 ng/mL for PrP and 487.83 ng/mL for MeP)^35^. These values are clearly higher than levels observed in the present study, but it should be remembered that urine is a completely different matrix than hair, and besides, the commercially available distillate is subjected to processing,during which it could be contaminated with PBs as preservatives. The second publication of PBs in cows concerns a raw milk, in which MeP (in concentration levels 0.21–0.38 ng/mL), EtP (0.08–1.40 ng/mL), PrP (< LOD-0.24 ng/mL) and BuP (< LOQ–0.10 ng/mL) have been found^33^. Therefore, PBs concentrations observed in a raw milk differ significantly from those obtained in this study. However, it should be noted that such comparison is very difficult, due to the fact that milk as a matrix is significantly different from the hair, milk analysis were performed in a completely different part of the world and the number of milk samples included in described study was very limited (n = 5)^33^. Other previous studies on PBs concentration levels in cow milk concern processed milk^58,59^ and as such cannot be used to assess the environmental exposure of cows to these substances.

The difficulties in comparison of PBs levels in the hair samples with values noted in other parts of organism result from the fact that chemicals entering the body are in varying degrees distributed, accumulated and metabolized in various tissues. These processes depend both on the route of exposure, substance structure and the species of animal. The experiments on rats have shown that dependences between the route of exposure, as well as the exact chemical structure and tissue distribution, pharmacokinetics and excretion from the organism also apply to PBs^60^. It should be pointed out that previous studies conducted on a wide range of species of fish, birds and mammals have shown various levels of PBs (depending on the tissue and species studied) in various internal organs, including liver, kidneys, muscles, gastrointestinal tract, testis and ovary^26,61^. Other studies have reported the presence of PBs in the amniotic fluid, placenta and livers of fetuses, whose mothers were exposed to PBs^62^. It is also known that PBs are present in the brain of humans^63^ and animals^26^, what suggests the ability of these substances to penetrate the blood–brain barrier. BPs have also been described in human adipose^64^ and breast tissues^65^, as well as in the hair (Table 2) and fingernails^66^. Till now tissue distribution and pharmacokinetics of PBs in cows are unknown, but by analysis of investigations concerning other substances with apolar structures (like PBs other than MeP and EtP), it can be assumed that PBs levels are different in various tissues and also depend on the animals’ gender and age^67,68^. However, previous studies on PBs in the human hair (Table 2) have indicated that hair samples are suitable for studies on both polar and apolar BPs.

Due to the fact that some previous studies have shown a relationship between the content of PBs and the age of people or animals^31,69^, such observations were also made during this study. In humans clear differences between PBs in younger and older persons are explained by different habits and different frequency of using cosmetics and personal care products, i.e. factors that are of no importance in the case of animals^17,69^. In turn, in animals such differences are less visible and may result from various hormonal activities and age-dependent discrepancies in metabolic processes^31^. Present results are not clear. Admittedly, the mean concentration levels of MeP in older animals were slightly higher, but without statistically significantly differences. This fact may suggest that differences noted previously in humans^69^ mainly result from factors connected with the human lifestyle.

The question arises, whether PBs in doses noted in the present study may negatively affect the health status of cows and be dangerous for consumers of products of animal origin. Unfortunately, based on the current state of scientific knowledge, the answer to this question is practically impossible. Firstly, toxic activity of PBs and their doses, which indeed may be dangerous for humans and animals are still discussed, although more and more investigations present harmful properties of these compounds^11^. Secondly, there is no information about metabolism of PBs in ruminants, and therefore about correlations between PBs levels in the hair and in serum, internal organs or milk. It should be pointed out that correlation between levels in PBs and other endocrine disruptors in the hair and other matrices is not quite clear even in humans^29,37^ and elucidation of this issue requires further comprehensive studies. On the other hand some studies have reported that PBs may negatively affect various organs even in low doses^70,71^. Not without significance is the fact that PBs are not the only endocrine-disrupting chemicals polluting the environment. They usually occur with a wide range of other agents showing synergistic effects^72,73^. In such situation even low degree of exposure to particular compound may have negative impact on living organisms^74,75^. It should be pointed out that previous studies have reported that MeP, which was observed in the largest percentage of samples included in this study, has a less harmful effect on living organisms than other PBs and practically does not show an acute toxicity^76^. So it would seem that its adverse effect on humans and animals is insignificant. However, more recent investigations have found that chronic or subchronic exposure to MeP may affect various internal organs and systems (including liver, cardiovascular and neuronal systems) and shows carcinogenic properties^77–79^. Moreover, some toxic activities of MeP are visible even at low environmentally concentrations of this substance^77,79^. These facts, together with the widespread occurrence of MeP in the environment, suggest that this substance may be harmful to living organisms.

Taking this into account, it cannot be excluded that PBs in concentration levels noted in the present study may have negative effects on animal health and penetrate products of animal origin, such as meat, offal or milk.

## Materials and methods

### Reagents

During the present study the following reagents were used: MeP, EtP, PrP, BuP, BeP (all ≥ 99%), and ammonium acetate (≥ 98%) were purchased from Sigma-Aldrich (St. Louis, MO, USA), methanol and acetonitrile (LC–MS grade) obtained from Fisher Chemical and phenobarbital used as internal standard (IS) from Lipomed AG, (Arlesheim Switzerland). Ultrapure water was produced by Merck’s Direct-Q 3UVwater purification system (Darmstadt, Germany).

### Sample collection

**Figure 3:**
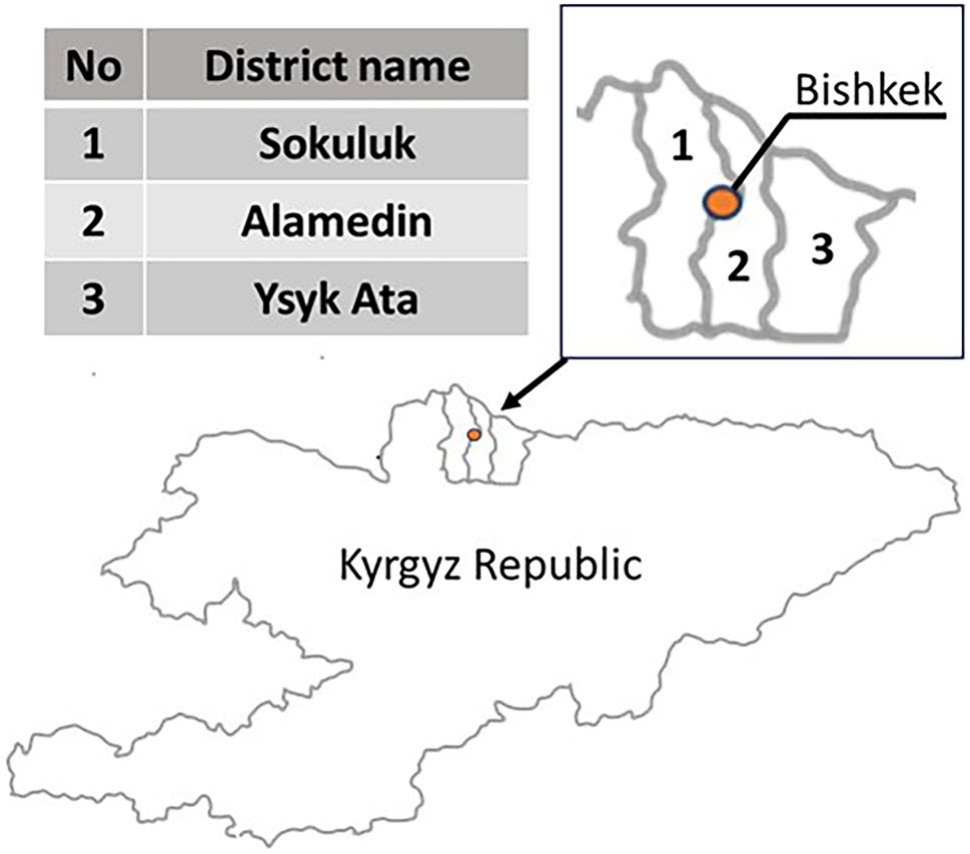
Localization of districts included into the study.

The present study included 48 adult dairy cows of various breeds in the age from 3 to 8 years old kept at farms located in three districts of the Kyrgyz Republic (description of animals is presented in Supplementary Materials (Table S2). Due to the fact that PBs first of all are present in the environment of the industrialized and urbanized regions the study involved cows from districts located in the vicinity of Bishkek—the capital and largest city of Kyrgyzstan, i.e. Sokuluk, Alamedin and Ysyk Ata (Fig. 3). The characteristics of the regions included into the study are presented in Table 3.

**Table 3:** Characteristic of regions included into the study.

No.	Name district	Distric human population	District area (km^2^)	District human population density (persons/km^2^)	Number of villiges	Places of sampling (name/coordinates)	Distance in the straight line from the center of Bishkek (km)
1	Sokuluk	147,208	1593	92	50	Sarban 42° 47ʹ 03.5ʹʹ N 74° 30ʹ 48.2ʹʹ E	10.32
						Kashka-Besh 42° 48ʹ 49.3ʹʹ N 74° 30ʹ 26.3ʹʹ E	8.64
2	Alamedin	158,137	2550	62	68	Kyzyl-Birdik 42° 42ʹ 49.7ʹʹ N 74° 42ʹ 47.6ʹʹ E	18.55
						Tash-Moinok 42° 44ʹ 21.9ʹʹ N 74° 39ʹ 21.4ʹʹ E	15.72
3	Ysyk Ata	131,503	2415	54.5	58	Kant 42° 53ʹ 00.2ʹʹ N 74° 50ʹ 39.5ʹʹ E	23.02

Hair samples were collected in April and May 2023. About 2 g of hair were cut closest to the skin from the same place (from the back above the shoulder blades) from each animal included into the study. Immediately after collection, hair samples were wrapped in aluminium foil and stored in the dark at room temperature until further analysis. During sampling and storage hair samples had no contact with items, which may contain PBs.

Due to the fact that hair samples collection was not painful or stressful and was performed during care and/or breeding activities, the consent of the ethics committee was not required under the laws in force in the Kyrgyz Republic and European Union. An informed consent to sample collection was obtained from the owners of animals included into the study. The authors complied with the ARRIVE (Animal Research: Reporting of In Vivo Experiments) guidelines.

### Paraben extraction

The hair was cut into small fragments with a length of several millimeters. Then, to remove any external contamination, hair was rinsed three times with ultrapure water and twice in methanol and dried at 50 °C. After the rinsing the extraction was performed in accordance with the method described by Tzatzarakis et al.^80^ and Wojtkiewicz et al.^24^. Shortly, 100 mg of hair sample with 2 × 2 ml of methanol and 25 ng IS in glass screw tubes were extracted in an ultrasonic water bath for 2 × 2 h with periodic mixing with a vortex system. The obtained extracts were combined and evaporated to dryness under nitrogen steam at 35 °C. After adding 100 μl of methanol to the residues, the solution was transferred into 2 ml vials with inserts for liquid chromatography-mass spectrometry (LC–MS) analysis, and 10 μl of the solution was injected into the system.

### Instrumentation

The analysis was performed with a LC–MS system (Shimadzu, Kyoto, Japan, LC–MS 2010 EV).A Supelco Discovery column C18 (250 mm, 4.6 mm, 5 μm; Sigma-Aldrich, St. Louis, MO, USA) was used to separate the analytes at a temperature of 30 °C. The analysis was made up with a flow rate of 0.6 ml/min using 5 mM ammonium acetate as solvent A and acetonitrile as solvent B. To monitor the aforementioned substances, an atmospheric pressure chemical ionization (APCI) and a quadrupole mass filter in negative selected ion monitoring (SIM) mode were used with ions m/z 151.05, 194.0 for MeP, 165.05, 208.1 for EtP, 179.0, 222.05 for PrP, 227.1, 270.1 for BeP, 193.05, 236.05 for BuP and 231.05 for the IS (Table 2)**.** The interface, curved desolvation line (CDL), and heat block temperatures were set at 400 °C, 200 °C, and 200 °C, respectively; the detector voltage at 1.5 kV; and the nebulizing gas flow at 2.5 L/min. The standard addition method was used for quantification.

### Method validation

Analytical parameters to evaluate the efficacy of the methods used were tested as follows. The linear response of the system was checked by standard solutions of the analytes at concentrations 0, 25, 50, 100, 250, and 500 ng/ml. Spiked hair sample analysis was performed for concentrations of 0, 25, 50, 100, 250 and 500 pg/mg with linearity from 0.9911 (for MeP) to 0.9991 (for BuP). Both limit of detection (LOD) and limit of quantification (LOQ) were evaluated using the signal to noise ratio. Spiked samples were used for the evaluation of the recovery, accuracy, and inter-day precision (%RSD) of the method at levels 25, 50, 100, 250 and 500 pg/mg. The mean % recovery values of the method were ranged from 75.5% for BeP to 117.8% for MeP. The corresponding values of % accuracy were ranged from 96.8% for EtP to 103.2 for MeP while the precision of applied methodology (%RSD) was calculated from 7.6% for PrP to 22.42% for BuP (Table 4).

**Table 4:** Validation parameters of the applied methodology.

	MeP	EtP	PrP	BeP	BuP
Rt (min)	14.1	15.9	17.6	19.05	19.1
Target m/z	151.05	165.05	179.0	227.10	193.05
Q m/z	194.00	208.10	222.05	270.10	236.05
% Recovery	117.8	82.2	93.6	75.5	79.3
n	5	5	5	5	5
% Accuracy	103.2	96.8	98.1	99.1	97.9
n	5	5	5	5	5
Precision (%RSD)	13.8	18.0	7.6	21.9	22.2
n	3	3	3	3	3
LOD	2.8	3.9	2.2	2.0	2.2
LOQ	9.4	12.8	7.4	6.5	7.5
n	3	3	3	3	3

*MeP* methyl paraben, *EtP* ethyl paraben, *PrP* propyl paraben, *BuP* butyl paraben, *BeP* benzyl paraben.

### Statistical analysis

The statistical analysis was performed using GraphPad Prism version 9.2.0 (GraphPad Software, San Diego, California USA). In characterization of data the descriptive statistics with calculation of arithmetic mean ± standard deviation (SD), median and frequency of detection has been used. In the case of a comparison of MeP levels between districts a non-parametric Kruskal–Wallis test was used, and in comparison of MeP levels between younger (at the age of 3–4 years) and older (at the age 5–8 years) animals a Mann–Whitney test was used. The differences were considered statistically significant at *p* < 0.05.

## Conclusions

To sum up, this study strongly suggests that the analysis of hair samples may be useful to determine the degree of exposure of cows to PBs, especially in the case of studies on long-term exposure It has also been shown that dairy cows in Kyrgyzstan are mainly exposed to MeP, and the concentration levels of this substance vary significantly between particular animals, even those living in the same area. It is in agreement with previous studies in various regions of the world, which also describes MeP as a dominant paraben in the environment and biological samples. Moreover, some correlations between the degree of urbanization and industrialization and MeP concentration levels in cow hair samples have been found. However, due to the fact that PBs metabolism in ruminants and correlations between BPs levels in the hair and other parts of organism are not clear, it is difficult to assess the impact of BPs in levels observed during this study on animal health or safety of milk consumers. Therefore, a lot of aspects connected with dairy cows exposure to BPs require further comprehensive research.

### Supplementary Information

Supplementary Tables.

## Data Availability

All data generated or analyzed during this study are included in this published article (and its Supplementary Information files).
