# Supplementary material for: Exposure assessment of dairy cows to parabens using hair samples analysis
Source: Sci Rep. 2024 Jun 21;14:14291. doi: 10.1038/s41598-024-65347-z (PMC11192892; doi:10.1038/s41598-024-65347-z)
Supplement: Supplementary file 1 — Supplementary Tables. [file 41598_2024_65347_MOESM1_ESM.docx]

**Supplementary materials to:**

**Assessment of exposure of dairy cows to parabens using analysis of hair samples**

Slawomir Gonkowski^1^, Manolis Tzatzarakis^2^, Naeiste Kadyralieva^3^, E. Vakonaki^2^, T. Lamprakis^2^

^1^ Department of Clinical Physiology, Faculty of Veterinary Medicine, University of Warmia and Mazury in Olsztyn, Oczapowskiego 13, 10-957 Olsztyn, Poland.

^2^ Laboratory of Toxicology, School of Medicine, University of Crete, 71003 Heraklion, Crete, Greece.

^3^ Kyrgyz-Turkish Manas University, Veterinary Faculty, Department of Histology and Embryology, Bishkek, Kyrgyzstan

**Corresponding author:** S. Gonkowski ,

e-mail: [slawomir.gonkowski@uwm.edu.pl](mailto:slawomir.gonkowski@uwm.edu.pl)

Table S1 Concentration levels of parabens (pg/mg) in particular animals including in the study

|  |  | **Concentration (in pg/mg)** | | | | |
| --- | --- | --- | --- | --- | --- | --- |
| **Sample No** | **Distric name/village name** | **MeP** | **EtP** | **PrP** | **BeP** | **BuP** |
| **1** | Sokuluk/Sarban | 111,8 | 14,6 | 9,7 | ND | ND |
| **2** | Sokuluk/Sarban | 42,1 | ND | ND | ND | ND |
| **3** | Sokuluk/ Sarban | 40,9 | ND | ND | ND | ND |
| **4** | Sokuluk/ Sarban | 50,8 | 13,0 | 10,5 | ND | ND |
| **5** | Sokuluk/ Sarban | 75,4 | 38,8 | 27,1 | ND | 7,6 |
| **6** | Sokuluk/ Sarban | 21,2 | ND | ND | ND | ND |
| **7** | Sokuluk/ Sarban | 49,5 | ND | ND | ND | ND |
| **8** | Sokuluk/ Sarban | 52,2 | ND | ND | ND | ND |
| **9** | Sokuluk/Kashka-Besh | 30,2 | ND | ND | ND | ND |
| **10** | Sokuluk/ Kashka-Besh | 59,3 | ND | ND | ND | ND |
| **11** | Sokuluk/ Kashka-Besh | 56,5 | ND | ND | ND | ND |
| **12** | Sokuluk/ Kashka-Besh | 67,7 | ND | ND | ND | ND |
| **13** | Sokuluk/ Kashka-Besh | 59,1 | 19,0 | ND | ND | ND |
| **14** | Sokuluk/ Kashka-Besh | 60,9 | ND | ND | ND | ND |
| **15** | Sokuluk/ Kashka-Besh | 41,5 | ND | ND | ND | ND |
| **16** | Sokuluk/ Kashka-Besh | 41,8 | ND | ND | ND | ND |
| **17** | Alamedin/Kyzyl-Birdik | 40,8 | ND | ND | ND | ND |
| **18** | Alamedin/ Kyzyl-Birdik | 36,1 | ND | ND | ND | ND |
| **19** | Alamedin/ Kyzyl-Birdik | 47,8 | ND | ND | ND | ND |
| **20** | Alamedin/ Kyzyl-Birdik | 48,1 | ND | ND | ND | ND |
| **21** | Alamedin/ Kyzyl-Birdik | 54,6 | ND | 9,1 | ND | ND |
| **22** | Alamedin/ Kyzyl-Birdik | 35,4 | ND | <LOQ | ND | ND |
| **23** | Alamedin/ Kyzyl-Birdik | 266,4 | ND | ND | ND | ND |
| **24** | Alamedin/ Tash-Moinok | 36,3 | ND | ND | ND | ND |
| **25** | Alamedin/ Tash-Moinok | 44,8 | ND | ND | ND | ND |
| **26** | Alamedin/ Tash-Moinok | 49,7 | ND | ND | ND | ND |
| **27** | Alamedin/ Tash-Moinok | ND | ND | ND | ND | ND |
| **28** | Alamedin/ Tash-Moinok | 118,7 | ND | ND | ND | ND |
| **29** | Alamedin/ Tash-Moinok | 76,5 | ND | ND | ND | ND |
| **30** | Alamedin/ Tash-Moinok | 109,7 | ND | ND | ND | ND |
| **31** | Alamedin/ Tash-Moinok | 69,3 | ND | 10,1 | ND | ND |
| **32** | Ysyk Ata/Kant | 365,5 | ND | ND | ND | ND |
| **33** | Ysyk Ata/ Kant | 18,3 | ND | 9,7 | ND | ND |
| **34** | Ysyk Ata/ Kant | 87,1 | ND | 10,8 | ND | ND |
| **35** | Ysyk Ata/ Kant | 42,8 | ND | ND | ND | ND |
| **36** | Ysyk Ata/ Kant | 34,5 | ND | ND | ND | ND |
| **37** | Ysyk Ata/ Kant | 39,6 | ND | ND | ND | ND |
| **38** | Ysyk Ata/ Kant | 30,2 | ND | ND | ND | ND |
| **39** | Ysyk Ata/ Kant | 12,0 | ND | ND | ND | ND |
| **40** | Ysyk Ata/ Kant | 22,3 | ND | ND | ND | ND |
| **41** | Ysyk Ata/ Kant | 23,2 | ND | ND | ND | ND |
| **42** | Ysyk Ata/ Kant | 24,9 | ND | ND | ND | ND |
| **43** | Ysyk Ata/ Kant | <LOQ | ND | ND | ND | ND |
| **44** | Ysyk Ata/ Kant | 28,2 | ND | ND | ND | ND |
| **45** | Ysyk Ata/ Kant | ND | ND | ND | ND | ND |
| **46** | Ysyk Ata/ Kant | 45,5 | ND | ND | ND | ND |
| **47** | Ysyk Ata Kant | ND | ND | ND | ND | ND |
| **48** | Ysyk Ata Kant | 66,2 | ND | ND | ND | ND |

ND-not detected, LOQ- limit of quantification

Table S2 Characterization of animals included into the study

| Sample No | Date of sampling | District name | Breed | Age (years) |
| --- | --- | --- | --- | --- |
| 1 | 13.04.2023 | Sokuluk | Alatau | 5 |
| 2 | 13.04.2023 | Sokuluk | Alatau | 6 |
| 3 | 13.04.2023 | Sokuluk | Swiss | 4 |
| 4 | 13.04.2023 | Sokuluk | Alatau | 8 |
| 5 | 13.04.2023 | Sokuluk | Alatau | 4 |
| 6 | 13.04.2023 | Sokuluk | Swiss | 6 |
| 7 | 13.04.2023 | Sokuluk | Swiss | 4 |
| 8 | 13.04.2023 | Sokuluk | Alatau | 6 |
| 9 | 04.05.2023 | Sokuluk | Swiss | 3 |
| 10 | 04.05.2023 | Sokuluk | Swiss | 3 |
| 11 | 04.05.2023 | Sokuluk | Swiss | 3 |
| 12 | 04.05.2023 | Sokuluk | Swiss | 3 |
| 13 | 04.05.2023 | Sokuluk | Swiss | 3 |
| 14 | 04.05.2023 | Sokuluk | Swiss | 3 |
| 15 | 04.05.2023 | Sokuluk | Swiss | 3 |
| 16 | 04.05.2023 | Sokuluk | Swiss | 3 |
| 17 | 12.05.2023 | Alamedin | Kyrgyz breed | 4 |
| 18 | 12.05.2023 | Alamedin | Kyrgyz breed | 7 |
| 19 | 12.05.2023 | Alamedin | Kyrgyz breed | 6 |
| 20 | 12.05.2023 | Alamedin | Kyrgyz breed | 4 |
| 21 | 12.05.2023 | Alamedin | Kyrgyz breed | 6 |
| 22 | 12.05.2023 | Alamedin | Kyrgyz breed | 4 |
| 23 | 12.05.2023 | Alamedin | Kyrgyz breed | 5 |
| 24 | 12.05.2023 | Alamedin | Kyrgyz breed | 6 |
| 25 | 12.05.2023 | Alamedin | Kyrgyz breed | 5 |
| 26 | 12.05.2023 | Alamedin | Kyrgyz breed | 7 |
| 27 | 12.05.2023 | Alamedin | Kyrgyz breed | 6 |
| 28 | 12.05.2023 | Alamedin | Kyrgyz breed | 4 |
| 29 | 12.05.2023 | Alamedin | Kyrgyz breed | 5 |
| 30 | 16.05.2023 | Alamedin | Holstein | 4 |
| 31 | 16.05.2023 | Alamedin | Simental | 6 |
| 32 | 13.05.2023 | Ysyk Ata | Holstein | 4 |
| 33 | 13.05.2023 | Ysyk Ata | Holstein | 4 |
| 34 | 13.05.2023 | Ysyk Ata | Holstein | 4 |
| 35 | 13.05.2023 | Ysyk Ata | Holstein | 4 |
| 36 | 13.05.2023 | Ysyk Ata | Holstein | 4 |
| 37 | 13.05.2023 | Ysyk Ata | Holstein | 4 |
| 38 | 13.05.2023 | Ysyk Ata | Holstein | 4 |
| 39 | 13.05.2023 | Ysyk Ata | Holstein | 4 |
| 40 | 13.05.2023 | Ysyk Ata | Holstein | 4 |
| 41 | 13.05.2023 | Ysyk Ata | Holstein | 4 |
| 42 | 13.05.2023 | Ysyk Ata | Holstein | 4 |
| 43 | 16.05.2023 | Ysyk Ata | Holstein | 4 |
| 44 | 16.05.2023 | Ysyk Ata | Holstein | 4 |
| 45 | 16.05.2023 | Ysyk Ata | Mixbreed | 4 |
| 46 | 16.05.2023 | Ysyk Ata | Holstein | 4 |
| 47 | 16.05.2023 | Ysyk Ata | Monbrew | 4 |
| 48 | 16.05.2023 | Ysyk Ata | Mixbreed | 4 |
